# Philippine Older Adults’ Attitudes to Love and Remarriage

**DOI:** 10.1093/geroni/igaa057.1453

**Published:** 2020-12-16

**Authors:** Ju Young Kim, Hanzhang Xu, Truls Ostbye, Grace Cruz

**Affiliations:** 1 Duke University, Durham, North Carolina, United States; 2 Duke University School of Medicine, Durham, North Carolina, United States; 3 Duke, Durham, North Carolina, United States; 4 University of the Philippines, Diliman, Quezon City, Philippines

## Abstract

Attitudes to love in older adults, often operationalized as acceptance of love and re-marriage in their 60s and 70s, is a key yet understudied component of aging in Southeast Asia. Using data from the 2007 Philippine Study on Aging that included 3105 older adults 60+, this study aimed to 1)describe the level of acceptance of love in older adults in the Philippines, 2)assess factors associated with acceptance of love, and 3)assess how acceptance of love is associated with social activity, life satisfaction, and health behaviors. Multivariate logistic regression was used to examine these associations while adjusting for age, gender, urban or rural residence, education, religion, marital status, self-reported health, comorbidity, and physical functioning). Only 1-in-5 older adults in the Philippines reported acceptance of love in older ages. Men and those with good health were more likely to report such acceptance, after adjustment for covariates (P<0.05). Although marital status alone had no association with acceptance of love, marital status interacting with gender showed significant associations with acceptance: unmarried men were more likely than married men to report acceptance. Individuals with lower acceptance of love were more likely to smoke (P<0.01). Attitude towards love was not significantly associated with social activity or life satisfaction after accounting for confounders. By evaluating the health and social outcomes associated with acceptance of love in older adults, this paper provided a better understanding of the utility of attitudes to love in older adults as a metric of elderly health in the Philippines.

